# HIV-1 Evolutionary Patterns Associated with Metastatic Kaposi's Sarcoma during AIDS

**DOI:** 10.1155/2016/4510483

**Published:** 2016-08-29

**Authors:** Susanna L. Lamers, Rebecca Rose, David J. Nolan, Gary B. Fogel, Andrew E. Barbier, Marco Salemi, Michael S. McGrath

**Affiliations:** ^1^Bioinfoexperts, LLC, 718 Bayou Lane, Thibodaux, LA 70302, USA; ^2^Department of Pathology and Laboratory Medicine, University of Florida, 2055 Mowry Road, Gainesville, FL 32610, USA; ^3^Natural Selection, Inc., 5910 Pacific Center Blvd., 6480 Weathers Place, San Diego, CA 92121, USA; ^4^The AIDS and Cancer Specimen Resource, University of California at San Francisco and the Department of Laboratory Medicine, Pathology, and Medicine, University of California at San Francisco, 1001 Poterero Ave, Bldg 3, Rm 207, UCSF Box 1317, San Francisco, CA 94110, USA

## Abstract

Kaposi's sarcoma (KS) in HIV-infected individuals can have a wide range of clinical outcomes, from indolent skin tumors to a life-threatening visceral cancer. KS tumors contain endothelial-related cells and inflammatory cells that may be HIV-infected. In this study we tested if HIV evolutionary patterns distinguish KS tumor relatedness and progression. Multisite autopsies from participants who died from HIV-AIDS with KS prior to the availability of antiretroviral therapy were identified at the AIDS and Cancer Specimen Resource (ACSR). Two patients (KS1 and KS2) died predominantly from non-KS-associated disease and KS3 died due to aggressive and metastatic KS within one month of diagnosis. Skin and visceral tumor and nontumor autopsy tissues were obtained (*n* = 12). Single genome sequencing was used to amplify HIV RNA and DNA, which was present in all tumors. Independent HIV tumor clades in phylogenies differentiated KS1 and KS2 from KS3, whose sequences were interrelated by both phylogeny and selection. HIV compartmentalization was confirmed in KS1 and KS2 tumors; however, in KS3, no compartmentalization was observed among sampled tissues. While the sample size is small, the HIV evolutionary patterns observed in all patients suggest an interplay between tumor cells and HIV-infected cells which provides a selective advantage and could promote KS progression.

## 1. Introduction

Prior to the human immunodeficiency virus (HIV) epidemic, Kaposi's sarcoma (KS) was considered a very rare disease, found primarily in Mediterranean countries and in organ transplant recipients [1]. Although Human Herpes Virus 8 (HHV8) is the etiological cause of KS [2], most HHV8^+^ individuals do not develop KS. Early in the HIV epidemic, KS was so frequently diagnosed that it was considered an AIDS-defining illness [3–5]. Prior to the mid-1990s and the use of combined antiretroviral therapy (cART), HIV^+^ patients had a 40% lifetime risk of developing KS [6]. Although combined antiretroviral therapy (cART) has undeniably reduced KS-associated mortality among HIV-infected patients [7–9], the statistical impact is unclear due to differing clinical definitions and cART adherence problems [10, 11]. Furthermore, KS is still one of the most common cancers among HIV^+^ patients [12] despite cART-improved CD4^+^ T-cell counts, low viral loads, and reduced risk of opportunistic infections [13–16]. KS is particularly problematic in resource-poor settings where cART and chemotherapy are limited [6, 17–19]; however, even in resource-rich settings, full remission of KS using cART and chemotherapy occurs in less than 50% of HIV^+^ patients with disseminated disease [20].

KS lesions can be solitary, localized, or disseminated, but the relationship among tumors in a single individual is largely unknown. While many KS lesions are cutaneous and indolent, tumors can occur in the oral cavity, lymph nodes, and viscera, which can result in fatal complications due to interference with normal body function. The growth of lesions can be very slow or explosively fast. The KS defining cell-type is a spindle cell (SC) [21, 22] that expresses markers of both endothelial and macrophage lineages [23]. KS tumors are polyclonal, positive for the presence of HHV8 virus, which infects the tumor-associated B-cells and SCs [23], and highly vascularized, giving them a distinguishing purple color. Inflammatory lymphocytes and monocytes/macrophages populate and enrich the KS tumor environment for tumor growth through production of a variety of cytokines [23]. These immune cells are all potential targets for HIV; however, to our knowledge, only one study assessed KS tumors for the presence of HIV using an early hybridization technique [24]. SCs can be cultured from PBMCs of patients with KS, suggesting that circulating SCs may lead to the appearance of multiple KS lesions [23].

HIV has the potential to infect and evolve in many anatomical tissues, including tumors [25–27]. Previously, we showed that the viral populations in lymphoma tumors were distinct from HIV in healthy tissues [25]. Here, our goal was to characterize HIV populations in KS tumors to determine whether HIV evolution could be used to track tumor relatedness and progression in native KS uncomplicated by the use of cART. We obtained tumor and nontumor tissues sampled* post mortem* from the AIDS and Cancer Specimen Resource (ACSR) from three HIV^+^/KS^+^ patients who died from aggressive AIDS, and we generated HIV* env-nef* sequences using single genome sequencing (SGS) to characterize viral evolutionary patterns.

## 2. Materials and Methods

### 2.1. Patients and Biomaterial

The three male patients included in this study were diagnosed with HIV infection and died with advanced AIDS without receiving cART (Table 1). 100 mg of frozen autopsy tumor and nontumor tissues was obtained through the ACSR (http://acsr.ucsf.edu/). Tissues were classified as tumor or nontumor by histological examination. The ACSR is a National Cancer Institute-funded tissue-banking program that obtains tissues from patients after appropriate consent and releases tissues to investigators following approval of the proposed study and deidentification of samples and clinical histories. The ACSR is recognized by the Office of Biorepositories and Biospecimen Research at the National Institutes of Health as being HIPAA compliant and in accordance with the ethical standards of the Declaration of Helsinki. Additionally, all material was obtained under approval from the University of California at San Francisco Committee on Human Research.

### 2.2. RNA/DNA Extractions

Total RNA and genomic DNA were isolated separately from each tissue section (30–50 ng) using AllPrep DNA/RNA Mini Kit (Qiagen #80204). Tissues were homogenized just prior to extraction in Buffer RLT Plus (lysis buffer) using a TissueRupter rotor-stator homogenizer (Qiagen #9001271) with a fresh sterile disposable probe (Qiagen #990890) for each sample. Manufacturer's guidelines were followed, with the exception of two 50 *μ*L final elutions using RNase-free water during the RNA isolation. The 100 *μ*L final volume of RNA generated was concentrated using RNeasyMinElute Cleanup Kit (Qiagen #74204). RNA and DNA extractions, cDNA synthesis, and first-round PCR setup were conducted in a restricted-access, amplicon-free room with separate air handling and laboratory equipment where no amplified PCR products or recombinant cloned plasmids were allowed, and work surfaces and equipment were thoroughly cleaned before and after use with Eliminase® (Decon Labs, Inc.). cDNA was synthesized using SuperScript® III First-Strand Synthesis System (Invitrogen Life Technologies #18080-051) using the provided Oligo(dT)_20_ primer according to manufacturer's recommendations. RNA was incubated at 65°C for 5 minutes with deoxynucleoside triphosphates (0.5 mM) and 5 *μ*M Oligo(dT)_20_ and then cooled quickly to 4°C. First-Strand cDNA Synthesis was performed in a 40 *μ*L reaction volume containing 1x reverse transcription buffer (10 mM Tris-HCl [pH 8.4], 25 mMKCl), 5 mM MgCl_2_, 10 mM dithiothreitol, 2 U/*μ*L of RNase-OUT*™* (RNase inhibitor), and 10 U/*μ*L SuperScript III RT. The reaction was heated to 50°C for 50 minutes, followed by 85°C for 5 minutes. The reaction was cooled to 37°C and 0.1 U/*μ*L of* E. coli *RNase H was added, followed by a 20-minute incubation. cDNA was stored at −20°C.

### 2.3. Sequencing Protocol

A modified single genome sequencing protocol was used to amplify linked HIV* env* and* nef* sequences [28]. cDNA and genomic DNA were serially diluted until an average of 30% or less of the nested PCR reactions were positive. During the first-round PCR, diluted cDNA or genomic DNA was amplified in 25 *μ*L reactions containing 1x Platinum® Blue PCR SuperMix (Invitrogen Life Technologies) and 0.2 *μ*M of each primer: EF2, 5′-ACAGTCTATTATGGGGTRCC-3′ and NR1, 5′-AGCTCCCAGGCTCAGATCT-3′ (6333–6352 bp and 9558–9576 bp of HIV HXB2, resp.) with the following cycling parameters: initial denaturation 95°C for 5 minutes, followed by 35 cycles of denaturing at 94°C for 1 minute, annealing at 58°C for 1 minute, and extension at 72°C for 4 minutes, with a final extension time of 10 minutes at 72°C. A second round of gp120 PCR consisted of 1 *μ*L of the first-round PCR added to a 24 *μ*L second-round reaction consisting of 1x Platinum Blue PCR SuperMix (Invitrogen Life Technologies) and 0.2 *μ*M of each primer: EF3, 5′ CATAATGTTTGGGCCACACA-3′ and ER2, 5′-CACCACTCTTCTYTTTGCC-3′ (6420–6439 bp and 7724–7742 bp of HIV HXB2, resp.) with the following cycling parameters: initial denaturation 95°C for 5 minutes, followed by 35 cycles of denaturation at 94°C for 1 minute, annealing at 58°C for 1 minute, and extension at 72°C for 2 minutes, with a final extension time of 10 minutes at 72°C. This second-round PCR generated a 1.3 Kb product, nearly covering the entire* env* gp120 gene. Second-round* env* gp120 PCR products were visualized on 1% agarose gels stained with ethidium bromide, and reactions containing a single 1.3 Kb product were considered positive and selected for sequencing. Subsequently, the first-round reactions that corresponded to positive second-round gp120 PCRs were then used to amplify the* nef* gene sequence. Second-round* nef* PCR consisted of 1 *μ*L of the first-round PCR added to a 24 *μ*L second-round reaction consisting of 1x Platinum Blue PCR SuperMix (Invitrogen Life Technologies) and 0.2 *μ*M of each primer: NF1, 5′-TTAGGCAGGGATAYTCACC-3′ and NR2, 5′-ATCTGAGGGCTCGCCACT-3′ (8347–8365 bp and 9488–9505 of HIV HXB2, resp.) with the following cycling parameters: initial denaturation 95°C for 5 minutes, followed by 35 cycles of denaturation at 94°C for 1 minute, annealing at 58°C for 1 minute, and extension at 72°C for 2 minutes, with a final extension at 72°C for 10 minutes. Second-round* nef* PCRs were visualized on 1% agarose gels stained with ethidium bromide, and reactions containing single 1.1 Kb products were considered positive and selected for sequencing. The primers were designed using Primer3 (http://bioinfo.ut.ee/primer3-0.4.0/) and by observing regions of conservation in alignments of published HIV subtype B sequences downloaded from Los Alamos HIV Sequence Database (http://www.hiv.lanl.gov/). Sequencing was performed on an Applied Biosystems 3730xl DNA Analyzer (Life Technologies) at the University of Florida Interdisciplinary Center for Biotechnology Research (UF ICBR). All sequences were assembled and aligned with the Geneious R7 software package (Biomatters http://www.geneious.com/) followed by a manual optimization of regions containing insertions and deletions. Sequence data has been submitted to GenBank (Accession numbers KU709129–KU709831).

### 2.4. Sequence Analysis

*Env* and* nef* alignments were generated using ClustalW [29] in MEGA5 [30] with further optimization performed by hand. Due to a large number of insertions and deletions that are typically problematic to align, regions with substantial insertions and deletions in* env* V1, V2, and V4 domains were removed. An initial maximum-likelihood phylogeny was inferred using sequences from all patients for both genes to ensure no cross-contamination. Pairwise distance analysis within and between RNA and DNA tissue sequence populations over time was assessed in MEGA5 [30] using the Tamura-Nei molecular model (identified using Model-Selection) with standard error estimated by 1000 bootstrap replicates. The Kruskal-Wallis test was used to determine significance between the mean pairwise distances in different anatomical tissues. Individual maximum-likelihood phylogenies were inferred for each patient under a GTR model of nucleotide evolution and gamma distributed rate variation among sites (+G) using PhyML [31]. Statistical support was assessed with 200 bootstrap replicates. The Slatkin-Maddison test [32] was used to determine compartmentalization between sequences derived from different tissues, and significance was assessed using 1,000 replicates of tip randomization, implemented in HYPHY v.1.0 [33]. The fast, unconstrained Bayesian approximations for inferring selection (FUBAR) model, implemented in Data Monkey (http://datamonkey.org/), were used to identify amino acid sites under selection in sequences derived from different tissues for each individual. Sites with posterior probability >0.9 of an increased (diversifying) or decreased (purifying) rate of nonsynonymous relative to synonymous substitutions were considered to have experienced a significant level of selective pressure. Selected sites were numbered according to the molecular HIV clone HXB2 (GenBank Accession #AF358141).

## 3. Results

### 3.1. Clinical Characteristics of the Patient Cohort

All patients (KS1–KS3) had CD4 counts <50 cells/mm^3^ at death and were diagnosed with KS only months prior to death (Table 1). KS1 lived with HIV infection for seven years. He was hospitalized due to* Mycobacterium avium* complex,* Pneumocystis pneumonia,* and wasting syndrome eleven months prior to death. Five months prior to death, medical notes indicate multiple AIDS-like symptoms; however, KS was not specified. At autopsy, numerous infections were identified throughout his body, including disseminated KS, which either had not been previously reported or had originated closer to death. KS2's clinical history is similar to that of KS1 in having multiple infections; however, this patient had an initial KS diagnosis five months prior to death. Also, this patient was diagnosed with an aggressive body cavity based lymphoma (BCBL) four months prior to death that was treated with chemotherapy. BCBL may be related to primary effusion lymphoma which is typically caused by HHV8, the Kaposi sarcoma herpes virus. KS3 had a nine-year history of HIV infection with reoccurring upper respiratory tract infections. He was diagnosed with KS one month prior to death. His KS lesions became rapidly disseminated on the skin and internally, which lead directly to his death due to respiratory distress.

### 3.2. HIV RNA and DNA Was Successfully Sequenced from All KS Tumors

Viral sequences were obtained from RNA in 13/14 tissues and from DNA in 12/14 tissues (Table 1). A preliminary maximum-likelihood (ML) phylogenetic tree of all sequences confirmed a distinct population of HIV sequences for each patient, indicating no laboratory cross-contamination.

### 3.3. KS Tumors Can Contain Distinct HIV Isolates

**Figure 1:**
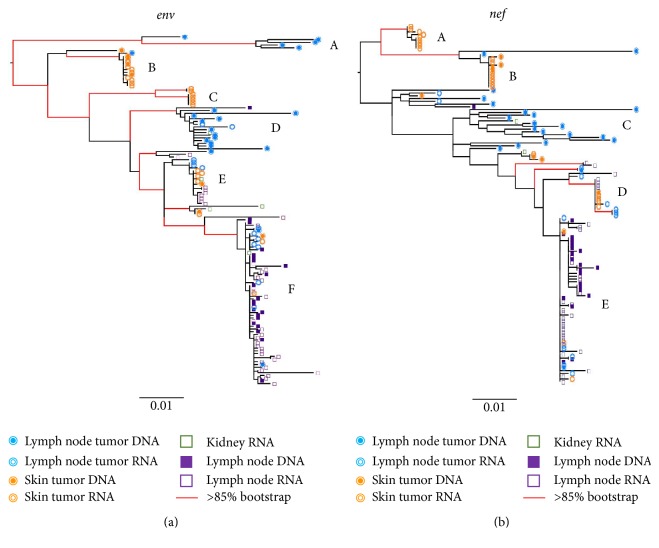
Maximum-likelihood phylogenies for KS1. Phylogenies were inferred for each patient using* env *(a) and* nef *(b) sequence alignments. Tips are labeled with symbols corresponding to the tissue of origin for each sequence. Branches with red lines indicate bootstrap values >85/200. Branches are scaled in substitutions/site according to the scale under each tree. Clades labeled A–F are discussed in the text.

**Figure 2:**
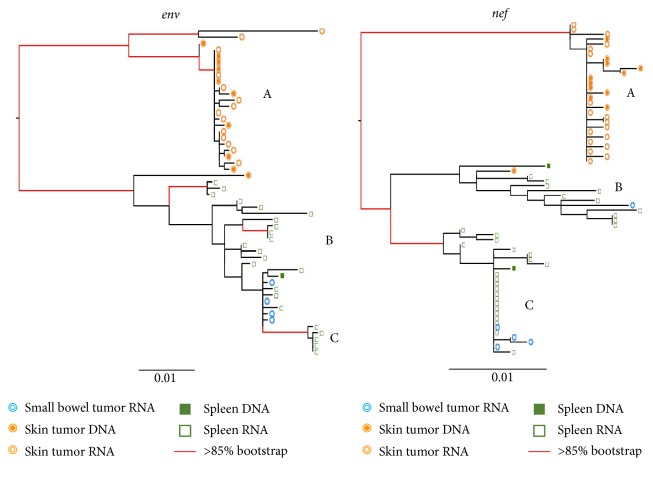
Maximum-likelihood phylogenies for KS2. See legend under Figure 1.

**Figure 3:**
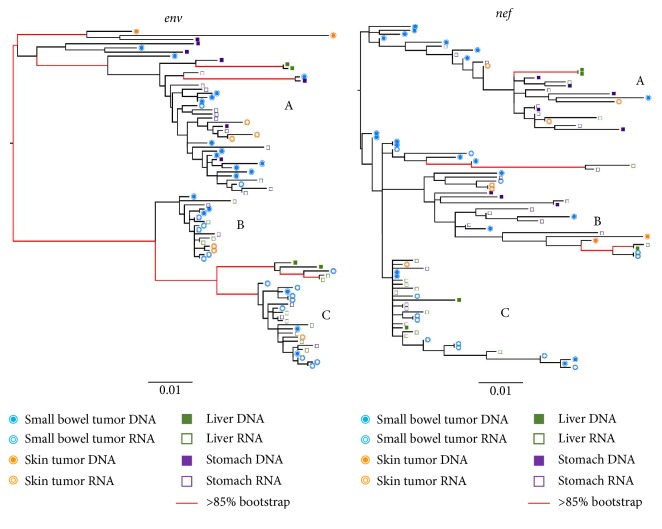
Maximum-likelihood phylogenies for KS3. See legend under Figure 1.

ML phylogenies were inferred for* env* and* nef* sequence populations for all three patients. In the KS1* env* phylogeny, four distinct and well-supported clades (A–D) were apparent that contained the majority of tumor-derived sequences (Figure 1). The relatively long internal branches leading to each clade indicate considerable divergence from the rest of the viral population. The majority of lymph node tumor sequences (19/34) were within two distinct clades (A, D). The majority of skin tumor sequences (20/32) were also located in two different distinct clades (B-C). One of these (B) contained both DNA and RNA sequences, while the other (C) was comprised exclusively of RNA skin tumor sequences. Most of the terminal branches leading directly to skin tumor-derived sequences were very short, while those leading to the lymph node tumor-derived sequences were considerably longer and showed more branching within the clade. The remaining lymph node and skin tumor RNA and DNA sequences were located in highly mixed clades (E-F). The* nef* phylogeny for KS1 had similar branching patterns, with even more pronounced diversity in one lymph node tumor clade (C). Similarly, all skin tumor-derived sequences except one clustered together in a distinct and well-supported clade in KS2 for both* env* and* nef* (A) (Figure 2). Small bowel tumor-derived RNA sequences were interspersed with the nontumor spleen-derived sequences in both genes; however, few bowel-derived sequences were obtained (*n* = 9). Branches in the nontumor clade showed considerable structure in both genes, consistent with a continuously diversifying virus population. In the* nef* phylogeny, two distinct clades were evident within the mixed clade, which was not present in the* env* tree. In contrast, for KS3, all tumor- and nontumor-derived sequences were largely interspersed in both* env* and* nef* phylogenies (Figure 3). Three well-supported clades were present in both genes (A–C). Interestingly, this patient was diagnosed with advanced and aggressive tumorigenesis less than one month prior to death.

### 3.4. KS-Associated Virus Is Compartmentalized in Tumors in KS1 and KS2 but Not in KS3

When pairwise distances were calculated for sequences derived from each tumor and nontumor tissue, only one significant difference was observed (*p* < 0.001) (KS1 lymph node tumor* nef* versus nontumor* nef* sequences). Viral populations were significantly compartmentalized within each of the two tumors and normal tissues for both env and nef in KS1 and KS2 (*p* < 0.001). On the other hand, gene flow among the three sites was found in both genes in KS3 (*env*: *p* = 0.021;* nef*: *p* = 0.171). Viral populations from combined tumor locations were again significantly compartmentalized compared with nontumor populations in* env* and* nef* in KS1 and KS2, as well as in* nef* for KS3 (*p* < 0.001).

### 3.5. Positively and Negatively Selected Codons Were Fewer and Tissue-Specific in KS1 and KS2 in Contrast to KS3 (Tables 2 and 3)

The proportion of codons under selection in* env* did not significantly vary between positively and negatively selected sites overall or within any single subject. However, the number of positions under either selective pressure was significantly higher in KS3 than in KS1 and KS2 (*p* < 0.01). In* nef*, a significantly higher number of negatively selected codons over positively selected codons were observed in all patients (*p* < 0.01). Codons under selection were tissue-specific in both KS1 and KS2, with only one being shared between two tissues in KS2. In contrast, in KS3* env* sequence populations, all shared positively and negatively selected codons in tumor tissues were also identified within nontumor sequences; similarly, in KS3* nef* sequence populations, all shared selected codons from tumors were identified in nontumor tissues except codon positions 31 and 67.

## 4. Discussion

In this study we examined if HIV evolutionary patterns could differentiate metastatic KS in patients untreated with cART. We analyzed HIV* env* and* nef* sequences from tumor and nontumor anatomical sites from three subjects who died with KS. Medical records indicated that subjects KS1 and KS2 had a slower KS progression than subject KS3; however, all patients had visceral KS, indicating an advanced cancer at death from AIDS. HIV RNA and/or DNA was amplified from all tumors in the study, demonstrating that, similar to a previous study [25], cancer tissues contain substantial amounts of HIV. Furthermore, HIV sequence populations from most tissues had similar genetic distances, indicating no constraints to HIV evolution in any of the tissue environments examined.

HIV from tumor sites in KS1 and KS2 was compartmentalized relative to the nontumor virus, with limited gene flow between compartments; however, subpopulations of virus were also present within tumors. Additionally, codon selection analysis demonstrated that, at the protein level, KS1 and KS2 had sequence populations evolving under independent selective pressures in different tumor/nontumor sites. In contrast, the patient who died from highly aggressive KS (KS3) showed remarkably different patterns. Here the virus in tumor and nontumor tissue was completely interspersed with a non-tissue-specific viral population structure. These findings could result from a number of scenarios, including the following: (1) subpopulations of virus in tumor could result from recent viral migration from another, unsampled anatomical location; (2) the viral population in the tumors is evolving independently due to a physical barrier restricting gene flow; (3) the viral populations in the tumors are evolving in response to local selective pressures; (4) viral sequences in one or more sites could represent archival virus that was reactivated because of the cancer growth. The first scenario is untestable here; however, we are conducting additional studies with more tumor and nontumor tissues from other KS patients, including KS derived from patients on cART with no detectable plasma viral load. While the second scenario is possible, unlike the brain in which viral flow is impeded by the blood-brain barrier, KS tumors are highly vascularized which would in theory provide ample opportunity for migration. Furthermore, a small number of tumor viruses were found in nontumor tissues, also suggesting a lack of physical barrier. In considering the third and fourth scenario, we did find that codon positions were under varied selective pressures in the tumors from KS1 and KS2 when compared to nontumor tissues (as expected from the phylogeny). For KS3, most, but not all, selected codons were shared among tissues; however, this patient had widely metastatic KS at the time of death. Therefore, it is possible that tumor-compartmentalized HIV populations could have existed in this patient prior to the rapid outgrowth and spread of the cancer, perhaps represented by one of the distinct subpopulations.

Reactivation and clonal expansion of viral populations could be consistent with the large clades of identical or nearly identical tumor-associated virus in KS1 and KS2 phylogenies and with the separation of DNA and RNA derived sequences into different clades within tumors of KS1. This hypothesis could be investigated further by identifying the infected cells types within each tumor. Furthermore, identifying the insertion sites within the host genome in these tumors could help understand if HIV-infected cells in tumors are physically migrating to other tumor sites. A recent HIV integration site study showed that one mechanism of HIV persistence within T-cells is related to clonal expansion [34]. Interestingly, the authors also found that insertion sites were enriched for genes involved in cell growth [34], suggesting that the insertion sites themselves may promote tumorigenesis. Studies within cancer and AIDS dementia tissue macrophages performed in our lab showed that major HIV integration sites in tissue-associated macrophages (TAMs) were related to cellular activation including those just upstream of the* c-fes* oncogene, which could contribute to clonal expansion [35–38]. This is consistent with a sequential pathogenesis model in which the insertion site of the virus promotes persistent cellular activation, clonal expansion, and migration of HIV^+^ from the tumor to nontumor sites [36].

The patients studied here were all cART-naïve; however, KS remains a substantial comorbidity even for patients undergoing cART. For some patients, rapid progression of KS occurs under cART even with an undetectable viral load [39]. These cases are frequently classified as related to immune reconstitution inflammatory syndrome (IRIS) [19, 39, 40], a well-studied phenomenon of resurgence of underlying pathologies during cART [41–46]. IRIS-related worsening of coinfections typically occurs within the first 6 months of therapy, is associated with localized tissue inflammation and low CD4 counts, and is most often observed in limited-resource settings [41, 47]. Although T-cell restoration and activation have been suggested as causing KS progression in these patients, no direct link has been demonstrated [47]. Innate immune dysfunction (e.g., macrophage-activation) has also been offered as an explanation for progressive KS during cART, whereby the interaction of restored T-cell populations with existing activated macrophages leads to increased IRIS [19].

## 5. Conclusions

Kaposi's sarcoma (KS) is a major cancer that still occurs in HIV positive patients despite immune-restorative combined antiretroviral therapy (cART). In this study we isolated HIV from tumor and nontumor tissue sites from patients who died with KS which had metastasized to the viscera (KS1–KS3). KS1 and KS2 died from other AIDS-associated complications, whereas KS3 died directly due to a highly aggressive KS that metastasized to lungs and caused death within 1 month of initial KS diagnosis. We compared the HIV sequences among patient's tissue sites and found that KS1 and KS2 had unique HIV in tumors compared to nontumor sites, whereas HIV was freely migrating among all tissues in KS3. While the study is based on a very small sample size, the results suggest that an HIV-associated mechanism may promote the metastatic process in aggressive KS. It is also important to note that it is unclear how the other AIDS-associated cancers may have impacted the study (e.g., lymphoma in KS1 and BCBL in KS2). Continued studies of the evolutionary patterns within and among KS tumors could help in understanding the mechanisms of metastasis as well as describing an additional HIV reservoir during cART. Evolutionary studies of HIV in tumor tissues are sorely lacking, and further investigation could provide additional clarity into the mechanisms of HIV-associated cancers.

## Figures and Tables

**Table 1:** Patient clinical history.

Patient	Age^1^	Major comorbidities^2^	KS location	Time since KS diagnosis (months)	Length of HIV infection (years)	Anatomical location of biopsy	Concentration (ng/*μ*L)		Number of *env* sequences		Number of *nef* sequences	
							RNA	DNA	RNA	DNA	RNA	DNA
KS1	34	Lymphoma, MAC, PCP, cytomegalovirus	Skin, pleura, lungs, trachea, lymph node, liver, adrenal glands	Unknown	7	KS skin	144	154	22	8	24	9
						KS lymph node	168	276	13	21	14	21
						Lymph node	120	12.1	37	22	35	26
						Kidney	>200	8.18	5	0	9	0

KS2	31	BCBL	Skin, pleura, lungs, esophagus, small bowel	~5	Unknown	KS skin	165	44	14	10	18	12
						KS small bowel	192	9.84	4	0	5	0
						Liver	191	8.42	0	0	0	0
						Spleen	190	13.9	23	1	31	2

KS3	28	Aggressive KS	Skin, pleura, lungs, trachea, esophagus, small bowel, colon, rectum, iliac lymph nodes, mesenteric lymph nodes, para-aortic lymph nodes, paratracheal lymph nodes	~1	9	KS skin	49	51	6	2	2	6
						KS small bowel	164	39.4	16	25	14	24
						Liver	161	33.4	14	5	15	5
						Stomach	78.2	40.2	24	8	20	8

^1^Age in years;  ^2^MAC: *Mycobacterium avium* complex; PCP: *Pneumocystis pneumonia*; BCBL: body cavity based lymphoma.

**Table 2:** Number (and percent) of codons under positive or negative selection in tumor and nontumor sites in *env* sequence populations.

Patient	Tissue	# positively selected sites (%)	Codon position of shared positively selected sites	# negatively selected sites	Codon position of shared negatively selected sites
KS1 (359)	Skin tumor	7 (1.9)	None	7 (1.9)	na
	Lymph node tumor	1 (0.3)	None	7 (1.9)	122
	Nontumor	None	None	8 (2.2)	122

KS2 (394)	Skin tumor	8 (2.0)	464	5 (1.2)	150
	Small bowel tumor	None	None	None	None
	Nontumor	5 (1.2)	373	4 (1.0)	150

KS3 (373)	Skin tumor	3 (0.8)	464	6 (1.6)	207, 247
	Small bowel tumor	10 (2.6)	183, 336, 396	16 (4.2)	103, 118, 207, 228, 238, 246, 254, 276, 374, 408, 415, 445
	Nontumor	11 (2.9)	143, 167, 183, 336, 454	21 (5.6)	103, 118, 207, 228, 238, 246, 254, 276, 374, 408, 415, 445

**Table 3:** Number (and percent) of codons under positive or negative selection in tumor and nontumor sites in *nef* sequence populations.

Patient	Tissue	# positively selected sites	Codon position of shared positively selected sites	# negatively selected sites	Codon position of shared negatively selected sites
KS1 (207)	Skin tumor	0	None	3 (1.5)	188
	Lymph node tumor	4 (1.9)	None	18 (8.7)	8, 37, 188
	Nontumor	1 (0.5)	None	4 (1.9)	8, 37, 188

KS2 (205)	Skin tumor	1 (0.5)	190	0	None
	Small bowel tumor	1 (0.5)	190	4 (2.0)	81
	Nontumor	2 (1.0)	190	5 (2.4)	81

KS3 (207)	Skin tumor	1 (0.5)	None	7 (3.4)	30, 65, 147, 151, 191, 195
	Small bowel tumor	1 (0.5)	None	14 (6.8)	4, 30, 63, 65, 111, 129, 151, 164, 191, 195
	Nontumor	1 (0.5)	None	21 (10.1)	4, 63, 111, 129, 147, 151, 164, 191, 195
